# Evaluation of Endovenous Laser Ablation in the Management of Varicose Veins

**DOI:** 10.7759/cureus.45096

**Published:** 2023-09-12

**Authors:** Nada Elzefzaf, Mohamed A Elfeky, Kareem M Elshatlawy, Ahmed Abdelal, Abdelaziz Elhendawy, Abdelrahman Ahmed, Mohamed Nada, Tarek Ouf

**Affiliations:** 1 Vascular Surgery, Manchester Foundation Trust, Manchester, GBR; 2 General and Colorectal Surgery, The Royal Oldham Hospital, Northern Care Alliance NHS Foundation Trust, Oldham, GBR; 3 Vascular Surgery, Menshawy General Hospital, Tanta, EGY; 4 Vascular Surgery, Alhada Military Hospital, Taif, SAU; 5 General Surgery, Tanta University Hospital, Tanta, EGY; 6 Vascular Surgery, Ain Shams University Hospitals, Cairo, EGY; 7 General and Colorectal Surgery, Ain Shams University Hospitals, Cairo, EGY

**Keywords:** peripheral vascular surgery, lower limb, radiofrequency ablation (rfa), endovenous laser ablation, varicose vein surgery

## Abstract

Background

The treatment of varicose veins has undergone tremendous changes over the years. High ligation of the saphenofemoral junction (SFJ) and stripping of the great saphenous vein (GSV) have been considered standard treatments for GSV insufficiency for over a century and are still adopted as the preferred method in the majority of surgical centers in North Africa. However, the increase in minimally invasive treatments such as endovenous thermal ablation (EVTA), radiofrequency ablation (RFA), ultrasound-guided foam sclerotherapy, and cryo-stripping has produced excellent results. Most patients who underwent these minimally invasive treatments were satisfied with their outcomes.

Methodology and results

In this clinical and prospective study, 30 cases (19 male and 11 female) of primary varicose veins underwent endovenous laser ablation (EVLA), and their outcomes were reviewed, and their results were satisfying to the patients. After EVLA with or without sclerotherapy, no major complications occurred (recurrence or recanalization) at the time of the study, although minor complications were quite common and included bruising or ecchymosis, postoperative pain that required analgesics, superficial thrombophlebitis, and skin burns that were very responsive to medical treatment.

Conclusion

Endovenous laser ablation continues to be a valid minimally invasive method for treating varicose veins with minimal complications and a very short recovery period, which appeals to patients.

## Introduction

The venous system of the legs acts as "a reservoir to store blood and as a conduit to return the blood to the heart" [1]. Blood is transported from the legs to the heart through a network of veins in the lower extremity. Compared with arteries, veins have thinner walls and "a weaker muscular layer and less elastic tissue," thus making them stiffer [2].

The leg comprises three distinct venous systems: deep, superficial, and perforating. Leg veins are categorized based on their position in relation to the muscular fascia, found in either the deep or superficial compartment [2]. Varicose veins are enlarged and twisted veins belonging to the superficial venous system [3].

In the past, saphenous varicose veins were mainly treated surgically by means of high ligation with or without stripping. Ultrasound-guided foam sclerotherapy (UGFS) has been used since 1997 and was the first minimally invasive treatment introduced. However, since 2000, endovenous thermal ablation (EVTA) using laser, radiofrequency, or steam has become more popular [4].

The endovenous laser ablation (EVLA) procedure is currently a well-established treatment modality for the entire incompetent great saphenous vein (GSV) segment. Laser energy is used to destroy the GSV in situ in combination with tumescent anesthesia [5]. Endovenous techniques have revolutionized the treatment of truncal varicose veins, and endovenous laser ablation has become the recommended first-line treatment method, achieving occlusion rates higher than 90% [6].

Endovenous laser ablation is thought to minimize morbidity after treatment compared with surgery, and it avoids groin incision and dissection at the saphenofemoral confluence, which has been reported to result in a lower complication rate and reduce post-treatment discomfort and pain, allowing a faster resumption of normal activity [7].

## Materials and methods

In this prospective study conducted from October 2017 to March 2018, a total of 30 patients (19 male and 11 female) seeking medical advice at various centers specializing in endovenous laser ablation were included. These patients presented with complaints of GSV varicosity, with or without reflux in the saphenofemoral junction (SFJ) and/or saphenopopliteal junction (SPJ). A detailed medical history, thorough clinical examination, and venous duplex ultrasound (DUS) scanning were performed on the enrolled patients. The study has been approved by the Faculty of Ain Shams University, General Surgery Department, Research Ethics Committee, Cairo, Egypt (approval number: 00006379).

Patient criteria

We have included patients in the age range of 20 to 60 years who presented with symptomatic primary varicose veins and a clinical, etiologic, anatomic, and pathophysiologic (CEAP) class C2-4EpAsPr (Ep(primary), As(superficial), and Pr(reflux)); GSV incompetence. Patients with recurrent varicose veins were also included if the GSV was preserved up to the groin on duplex imaging. Informed consent was obtained from all patients.

Our exclusion criteria included patients with small saphenous vein reflux, a history of arterial insufficiency, an ankle-brachial pressure index below 0.9, or both. Also, patients with tortuous GSV, rendering the vein unsuitable for endovenous treatment, were excluded.

The DUS examination was performed using the color-coded duplex Mindray system^TM^ for all patients before and after their therapy, using the same system or the laptop portable system of the same manufacturer in the operative session. The initial duplex scan was aimed at mapping the deep, superficial, and perforating venous systems while the patient was in a standing position.

The purpose of conducting preoperative DUS was to measure the size of the superficial venous system, identify venous reflux, and rule out any previous occurrences of deep vein thrombosis (DVT) or deep venous insufficiency. The postoperative color duplex ultrasound aimed to assess venous reflux, the presence of a thrombus, and recanalization in the ablated vein.

The success of the ablation procedure was determined by factors such as the inability to compress the treated segment of the GSV, the absence of blood flow inside the vein, a reduction in vein diameter, and the non-existence of deep venous thrombosis.

Operative technique

The patients were positioned in an anti-Trendelenburg posture on the table. During the procedure, the GSV was cannulated either at knee level in 10 patients using a percutaneous needle puncture under ultrasound guidance or at ankle level in other patients.

Endovenous laser therapy (EVLT) with a 1470 nm laser was performed on all 30 patients under tumescent anesthesia. A 4 Fr guide sheath was then advanced over a 0.035'' x 55cm j-tip guide wire. Once the correct placement of the sheath was confirmed, a laser fiber was inserted into the vein, with its distal tip positioned 2-3 cm distal to the SFJ. Tumescent anesthesia was administered once the device was appropriately positioned for ablation. The tumescent local anesthetic solution used was a mixture of 20 ml 1% lidocaine and 1 ml adrenaline (1:100000) diluted in 500 ml of cold (40 °C) saline.

Laser energy was subsequently delivered, and the laser fiber was slowly withdrawn under ultrasound guidance at a velocity of 3 mm/second until it reached a distance of 2/2.5 cm from the puncture site of GSV. Manual pressure was applied over the access site to obtain hemostasis. Thigh-level, class II graduated compression stocking was applied for two weeks.

The DUS criteria for successful treatment were established as follows: at one-week follow-up, an enlarged non-compressible GSV minimally decreased in diameter, with echogenic, thickened vein walls and no flow seen within the occluded vein lumen; at two- and six-month follow-ups, an occluded GSV with a substantial (50%) reduction in diameter was observed.

The vein lumen was usually obliterated by the thickened wall, which had low-level echoes and was incompressible. This wall thickening should be differentiated from acute GSV thrombosis, where the vein is also incompressible but the lumen is filled with an echoic acute thrombus. Several weeks after successful endovenous laser treatment, the resolution of the acute inflammation in the vein wall should result in a reduction in vein diameter.

Clinical evaluation was performed on all subjects at one week, two months, and six months. Patients were asked about symptomatic relief on follow-up visits, particularly regarding any improvement or resolution of lower-extremity pain associated with venous insufficiency.

Improvement in the appearance of the leg, including a reduction in visible varicosities, swelling, pigmentation, or other skin changes secondary to chronic venous insufficiency (CVI), was assessed by the patient and in direct comparison with pre-treatment photographs obtained from all subjects undergoing treatment. The patients were evaluated for possible adverse reactions caused by EVLT at each follow-up visit.

Minor complications were defined as those that had no significant clinical sequelae, such as bruising. Major complications were defined as those necessitating an increased level of care, surgery, or hospitalization.

## Results

Between October 2017 and March 2018, 30 patients (19 male and 11 female), aged 20-60 years old, presented with primary varicose veins of the lower limbs with or without ulcers, of which two were treated bilaterally. Cases were retrieved from those attending multiple centers performing EVLA in Cairo, Egypt. Written informed consent was obtained from all patients before participating in the study.

The intervention involved EVLA, sometimes combined with foam sclerotherapy, either during the same session or in subsequent follow-up visits. The gender distribution among the subjects was 37% female and 63% male. The demographic data of the participants and the number of affected limbs are presented in Table 1.

**Table 1:** Demographic data of the study participants N: number

Demographic data	Descriptive statistics
Age: Range, Mean ± SD	(20-60) 37.31034 ± 8.25429
Sex: N (%) Male, Female	19 (63%), 11 (37%)
Marital status: N (%) Single, Married	16 (53%), 14 (47%)
Treated limb: N (%) (N=32 limbs) Right, Left, Bilateral	20 (66.67%), 8 (26.66%), 2 (6.67%)

The studied patients were classified according to the CEAP classification. The clinical categories of the studied 30 patients are shown in Table 2.

**Table 2:** Clinical classification of the studied patients No.: number

Clinical categories	No. of affected limbs (N=32 limbs)	Percentage
C0: No visible venous diseases	0	0%
C1: Telangiectatic or reticular veins	0	0%
C2: Varicose veins	20	62.55%
C3: Varicose veins with edema	5	15.6%
C4: Varicose veins with skin changes without ulcer	5	15.6%
C5: Varicose veins with skin changes and healed ulcer	2	6.25%
C6: Varicose veins with skin changes and active ulcer	0	0%

The anatomical classification of varicose veins in the 30 studied patients (N = 32 limbs in 30 patients) is shown in Table 3.

**Table 3:** Anatomical classification of the studied patients GSV: great saphenous vein

Anatomical	Number of affected limbs	%
GSV along the whole length	27	(84.375%)
GSV above the knee	5	(15.625%)

Every treated patient in the study presented with symptoms related to their condition. The most prevalent symptom observed was visible varicose veins, which were present in nearly all patients. Additionally, other symptoms reported by the participants included pain, night cramps, restless leg sensations, bleeding, and skin discoloration (Table 4).

**Table 4:** The main symptoms in the studied patients

Symptoms	Number of patients	Percentage
Pain	30	(100%)
Visible varicose vein	28	(93.33%)
Night cramps	2	(6.67%)
Restless leg	15	(50%)
Bleeding	1	(3.33%)
Skin discoloration	5	(16.67%)

During the colored duplex examination, it was observed that all patients had incompetent saphenous veins in either one or both limbs, with significant reflux detected in the great saphenous veins. The examination further guided the selection of the puncture site for the GSV, either at the knee level or ankle level, based on the diameter of the vein, as indicated in Table 5.

**Table 5:** Duplex study of the studied patients No.: number; GSV: great saphenous vein

Duplex ultrasound	No. of the limbs (N=32 limbs in 30 patients)	Percentage
GSV reflux	32	(100%)
GSV puncture at the level of the ankle	27	(84.375%)
GSV puncture at the level of the knee	7	(21.875%)

Postoperative duplex scans were conducted on all 30 patients at four specific intervals: immediately after the operation, one week after, two months after, and six months after the procedure. The purpose of these scans was to evaluate both the superficial and deep venous systems. The results revealed a reduction in the diameter of the GSV in all limbs, except for one patient whose GSV diameter remained greater than 7 cm during the first-week follow-up duplex after the operation (Table 6).

**Table 6:** Postoperative duplex in the studied patients GSV: great saphenous vein; SFJ: saphenofemoral junction

Time	Diameter of the GSV: 3 cm below the SFJ	Diameter of the GSV: mid-thigh	No. of the limbs (N=32 limbs in 30 patients)	Percentage
Preoperative	11.5	7.8	32	100% both
One week postoperative	(7.6 +/- 1.00) mm	5.5	31	96.875%
Two months postoperative	(5.5 +/- 1.00) mm	4.5	31	96.875%
Six months postoperative	(3.5 +/- 1.00) mm	3.0	31	96.875%

All patients in this study underwent EVLA using tumescent local anesthesia. The laser energy administered is exhibited as joules (J)/cm vein. The energy was calculated based on the vein diameter. Table 7 shows a suggested parameter for 1470 nm endovenous laser ablation.

**Table 7:** Parameters of the laser beam GSV: great saphenous vein; LEED: linear endovenous energy density

GSV segment	Power of laser(w)	Pull-back rate (mm/s)	Energy delivered (LEED)(j/cm^2^)
Proximal 1/3 of GSV	14	1	140
Middle 1/3 of GSV	14	2	70
Distal 1/3 of GSV	14	3	47

Chemical sclerotherapy was administered to certain cases that displayed residual dilated tributaries after undergoing EVLT. Typically, this sclerotherapy procedure is carried out during the same session as the EVLT. The rate of injection for our patients is presented in Table 8.

**Table 8:** Sclerotherapy in the studied patients (N=32 limbs in 30 patients) GSV: great saphenous vein; EVLT: endovenous laser therapy

Type of varicose vein	Type of procedure	No. of limbs
GSV incompetence without dilated tributary	EVLT alone	25 (78.125%)
GSV incompetence with dilated residual tributaries	EVLT with injection sclerotherapy	7 (21.875%)

Following EVLA, with or without concurrent sclerotherapy, there were no major complications reported. However, minor complications were relatively common and included bruising or ecchymosis, postoperative pain necessitating analgesics, superficial thrombophlebitis, and skin burns, as indicated in Table 9.

**Table 9:** Postoperative complications of EVLT in the studied patients EVLT: endovenous laser therapy

Complication	Number of the patient	Percentage
Bruising	10	(33.3%)
Ecchymosis	5	(16.6%)
Pain	20	(66%)
Superficial thrombophlebitis	5	(16.6%)
Skin burn	1	(3.33%)
Recurrence	0	(0%)

## Discussion

For more than a decade, conventional surgical options for varicose veins in the form of high ligation or excision of the GSV were the leading trend. Long ago, medical professionals did not give much consideration to treating venous issues and only offered suffering patients conservative management. The major shift towards less invasive procedures in medicine also included varicose vein treatments, including but not limited to techniques such as radiofrequency ablation (RFA), EVLA, and foam sclerotherapy [8].

The pathophysiology of venous disease has shaped the progress of surgical management. The main driving force for pursuing better management of varicose veins was the decreasing recurrence rate and morbidity. High ligation of GSV, complemented by stripping of the vein length, has decreased recurrence, while minimally invasive procedures have added the value of less morbidity and fewer hospital stays [9].

Boné first reported on the delivery of endoluminal laser energy [10]. Since then, an EVLA procedure to treat the entire incompetent GSV segment has been described [11,12]. Endovenous laser therapy with a 980-nm diode laser system has been deemed a clinically safe, feasible, and well-tolerated technique that does not cause cosmetic problems in the setting of day-case surgery, allowing people to return to their normal daily activities rapidly [12]. Endovenous laser therapy, approved by the United States Food and Drug Administration in January 2002, introduced laser energy in the blood vessel lumen [12].

This study is focused on EVLT utilizing a 1470-nm diode laser system in the treatment of primary varicose veins. The study examined 32 limbs of 30 patients, and the mean follow-up period was six months. The patients, aged 20 to 60 years old, presented with primary varicose veins of the lower limbs with or without ulcers, of which two were treated bilaterally. The patients’ mean age was 37.3 years, and 19 (63%) male and 11 (37%) female patients were included in the study. However, the sample size of this study was smaller than that of recent studies done by Dzieciuchowicz, who examined 161 limbs in 154 patients, including RFA in 43 extremities, and followed up on his patients for 24 months [13]. Similarly, our study was smaller than Theivacumar's study, which treated a total of 95 patients (104 limbs) undergoing EVLA and followed them for 52 weeks [14].

The study "The first 1000 cases of the Italian Endovenous-Laser Working Group (IEWG)" by Agus GB et al. between 1999 and 2003 [7] was a multi-center clinical study where 1076 limbs were examined in 1050 patients, with a mean age of 54.5 years; 241 males (23%) and 809 females (77%) affected by CVI were considered eligible for surgery, and patients were then offered EVLA instead and classified by the CEAP classification over four years (January 1999-December 2003). Almost all patients had a DUS assessment at 36 months, with some cases lost to follow-up [7].

On one hand, our study was smaller in sample size and duration than a study done by J. Desmyttere et al., who followed up on 128 patients (147 limbs) for three years. Similarly, the present study was smaller than a study on 84 patients (96 limbs) for a one-year duration by S.W. Park et al. [15,16]. On the other hand, our sample size of patients and duration were similar to those of a study by Sadick et al., who followed up on 30 patients for 24 months [17].

In this study, the classification of varicose veins was based on the CEAP classification; the included patients were classified as follows: C2 was present in 20 limbs (62.55 %), C3 was present in five limbs (15.6%), C4 was present in five limbs (15.6%), and C5 was present in two limbs (6.25%). In a similar study, according to the clinical part of the same classification, chronic venous insufficiency patients were categorized as having C2 in 143, C3 in five, C4 in 12, C5 in two, and C6 in nine limbs [13].

N. S. Theivacumar treated a total of 104 limbs in 95 patients undergoing EVLA, who were categorized into four groups: Group GR (recurrent GSV reflux), Group GP (primary GSV reflux), Group SR (recurrent SSV reflux), and Group SP (primary SSV reflux) [14]. The details of the CEAP grades for each group were as follows: C2 in 27 (53%), 30 (59%), 10 (42%), and 11 (46%) limbs, respectively; C3 in nine (17.6%), seven (14%), seven (29%), and eight (33%) limbs, respectively; C4 in 10 (19.6%), 11 (21%), five (21%), and four (17%) limbs, respectively; and C5/6 in five (9.8%), three (6%), two (8%), and one (4%), respectively [14].

Agus et al. (2006) found that advanced CVI, defined as stage C4-C6, was present in only 84 (8%) of all patients. The rest of the patients were classified as C2-C3, which was the majority, with 861 patients (82%) in C2 and 105 (10%) in C4 [7]. In 36% (378 patients) of the total 1050 patients, the authors observed simultaneous clinical stages of CVI, with association C2-C3 in 270 (25.7%) patients, C2-C4 in 25 (2.3%) patients, and C2-C6 in 73 (6.9%) patients. A venous ulcer was found in only 11 patients (1.04%) [7].

Another study published by Michael Harlander-Locke et al., with a sample size of 80 patients, was classified according to the CEAP classification: 21 patients (26%) were in C2, 18 patients (23%) were in C3, five patients (6%) were in C4, four patients (5%) were in C5, and 32 patients (40%) were in C6. This study was more concerned with the outcome of EVLT in the management of venous ulcers, so the bulk of the sample was under C6 [18].

Myers & Jolley treated 361 patients with 509 saphenous veins in 494 limbs. All patients were compliant with follow-up for a long term of five years. There were 232 female (64%) and 129 male (36%) patients with an age range of 24-76 years (median age of 52 years) [19].

The CEAP classification showed that 449 limbs (91%) had uncomplicated varicose veins (C2, C3) and 45 limbs (9%) had complications (C4-C6) due to lipo-dermatosclerosis (n = 34), healed past venous ulceration (n = 5), or active ulceration (n = 6). Primary disease was present in all limbs, and none had features of the post-thrombotic syndrome [19].

The main presenting symptom in this study was aching pain, which was present in 30 patients (100%). Other symptoms included visible varicose veins in 28 patients (93.33%), night cramps in two patients (6.67%), restless legs in 15 patients (50%), bleeding in one patient (3.33%), and skin discoloration in five patients (16.67%). This was comparable to Campbell et al., who performed a study on 100 patients (151 limbs). Aching pain was also the predominant symptom present in 97 limbs (64%). Other variable symptoms included skin changes or eczema in 40 limbs (26%), cosmetic disfigurement in 32 limbs (21%), ankle edema in 32 limbs (21%), ulceration in 24 limbs (16%), heaviness in 18 limbs (12%), phlebitis in 10 limbs (7%), and bleeding in one limb (7%). Given that many patients reported more than one main symptom, the total percentage exceeded 100% [20].

All patients included in the study underwent duplex ultrasound examinations. Among the studied limbs, GSV reflux was identified in all 32 cases (100%). Dzieciuchowicz successfully managed 147 out of 185 (79.5%) GSVs, 23 (12.5%) small saphenous veins (SSVs), one (0.5%) Giacomini vein, eight (4.3%) anterior accessory saphenous veins (AASVs), and six (3.2%) dilated thigh tributaries of GSV in a total of 154 patients (comprising 171 limbs and 185 veins). In 14 limbs, more than one venous trunk was subjected to ablation. The distribution of treated veins did not show significant differences between the groups (p = 0.27) [13].

In the present study, a peri-venous application of the tumescent local anesthetic solution was performed, which consisted of 20 ml of 1% lidocaine and 1 ml of adrenaline (1:100000) diluted in 500 ml of cold (4°C) saline.

Adrenaline promotes vasoconstriction of the vein, thus reducing the incidence of hematoma and hyperpigmentation. Michael Harlander-Locke et al. administered an injection of tumescent anesthesia (saline, xylocaine, epinephrine, and sodium bicarbonate) using a 23-g spinal needle to the tissues surrounding the SSV along its entire length before ablation, all of which are done under ultrasound guidance. The procedure was carried out under local (tumescent) anesthesia in 140 (91%) patients in the study by Dzieciuchowicz et al.. The procedures were carried out under general anesthesia in nine patients (6%) and under spinal anesthesia in five patients (3%). In 148 patients (96%), a mini-phlebectomy was performed concurrently with the main procedure. [13].

The study utilized the advanced German Fox^TM^ and Chrolase^TM^ 1470-nm diode laser systems. The mean energy applied during the procedure was 70 J/cm. Laser energy was determined based on the GSV diameter, 1.5-2 cm distal to SFJ, using the linear endovenous energy density (LEED) values. For GSV diameters ranging from 4.5 to 6.9 mm, an energy of 60-70 J/cm2 was employed. In cases with GSV diameters of 7-10 mm, an energy of 80-90 J/cm2 was utilized. These energy values were comparable to those used in other studies by Theivacumar et al. (60-70 J/cm), Timperman et al. (63.4 J/cm), and Proebstle et al. (63 J/cm) [14, 21, 22].

Theivacumar et al. assessed factors influencing the effectiveness of EVLA in the treatment of GSV reflux at the Leeds Vascular Institute, The General Infirmary, Leeds, UK. The findings of their study form the basis for developing a standardized protocol for a successful EVLA. It is clear that an energy density (ED) ≥ 60 J/cm is central to achieving complete GSV occlusion. This equates to 5 pulses/cm vein when using 12 watts’ power, 1-second pulses, and 1-second intervals for laser fiber withdrawal, i.e., 2 mm pull back during each 1-second interval [14].

The choice of pulsed laser for EVLA was based on the description of the technique and the results reported by Min et al.. The principal benefit of continuous withdrawal is a reduction in procedure time and fewer complications. The ED (J/cm) of laser delivery is the main determinant of a successful GSV ablation following EVLA [12,14]. A lower failure rate was reported by Timperman et al. when an energy of around 63.4 J/cm was applied [21]. In contrast, Kim & Paxton achieved 100% technical success in 34 patients using a 980-nm diode laser at 11 W power, delivering 35.16 J/cm, reporting a lower incidence of superficial burns or palpable induration [23]. Finally, Proebstle et al. produced data suggesting that the energy delivery required to achieve reliable GSV ablation and low re-canalization rates is more dependent on the vein diameter than the quantity of energy delivered [22].

A marked reduction of symptoms associated with varicose veins in the form of improvement of edema, pain, swelling, and ulceration was noticed in the follow-ups of the studied patients. This finding was comparable to that of other studies, which also described the reduction of symptoms associated with varicose veins after EVLT [9,22].

At the end of the study period, the ablated veins appeared as a fibrotic, non-compressible remnant of the vein on the duplex scan. Our short-term clinical success reduced symptoms, markedly resolved varicose veins, and promoted ulcer healing. It also resulted in very high patient satisfaction within a year, with improved patient symptomatology, pain-free legs, and no disfigurement. These cumulative studies with EVLA were necessary to prove the superiority of minimally invasive techniques over traditional surgery, making it the treatment of choice [9].

While EVLT has shown several advantages, including lower recurrence rates compared with ligation and stripping and requiring minimal groin dissection, it also includes the ability to selectively ablate incompetent veins, thus preserving the venous drainage in the competent tributaries [24].

Postoperative pain had limited variability, ranging from soreness through discomfort to mild pain (score 3). In this study, 20 (66%) patients reported pain. Analgesics were given routinely to all studied patients. Sharif et al. reported that patients began feeling pain five to eight days after the intervention, mainly due to the inflammatory reaction related to the ablation and not damage to surrounding structures [25].

Chang & Chua studied 12 patients (15 limbs) who described no severe complications such as DVT, pulmonary embolism (PE), or skin burns at the entry point [26]. Unfortunately, in this study, only one case (3%) suffered from small skin burns, most probably due to inadequate administration of tumescent anesthesia.

Tolerable adverse effects were observed in the laser-treated region, which spontaneously resolved within three weeks. Bruising in 15 legs, induration in nine legs, and mild tenderness in eight legs were observed. One patient had superficial thrombophlebitis, which resolved in a week with a non-steroidal anti-inflammatory drug. There were no adverse effects related to the local anesthesia. Overall, bruising was the most common adverse effect, which was usually mild to moderate and subsided within one to three weeks [26].

Generally, complications of EVLA were well tolerated. Minor complications such as induration and ecchymosis occurred due to inflammation around the vein, which improved with a short course of an anti-inflammatory drug. Regarding the major complications, four out of the six recanalized veins were only partially open, and ultrasound-guided sclerotherapy was used to resolve them successfully [9]. The same happened in patients in the present study, where no major complications (no DVT, no PE, and no recurrence) were recorded in any of the 30 cases. However, after the EVLA ablation with or without sclerotherapy, minor complications were quite common and included bruising in 10 (33.3%) patients, ecchymosis in five (16.6%) patients, postoperative pain that required analgesia in 20 (66.67%) patients, superficial thrombophlebitis in five (16.6%) patients, and skin burn in one (3.33%) patient.

Our study was limited by the small number of patients that we were able to recruit. Furthermore, our patients were recruited from multiple centers; however, they were all from a single city, which may represent another limitation of our study.

Varicose veins are an extremely common problem in the local community of the study, and their treatment places a considerable strain on the medical system, with a long waiting time for operation in the public hospital system. Endovenous procedures allow more efficient management of large numbers of patients with outpatient treatment. Endovenous laser therapy is simple to perform, well-accepted by patients, and relatively atraumatic and safe.

## Conclusions

Endovenous laser ablation provides improved patient recovery in terms of markedly lower postoperative pain, a shorter hospital stay, and a lower incidence of bruising (hematomas). It avoids surgical incisions, mechanical disruption of the SFJ, and the aggressiveness of avulsion of the saphenous vein with overall tolerable post-operative complications.
